# Transurethral resection of the prostate in the extreme elderly (≥ 85 years): treatment success, morbidity and survival

**DOI:** 10.1007/s00345-025-05948-z

**Published:** 2025-09-24

**Authors:** Stephen Baug, Christian Beisland, Christian Arvei Moen, Per Odland, Jesper Blomquist, Patrick Juliebø-Jones

**Affiliations:** 1 Department of Urology, Haraldsplass Deaconess Hospital, Bergen, Norway; 2 Department of Urology, Haukeland University Hospital, Bergen, Norway; 3 Department of Clinical Medicine, University of Bergen, Bergen, Norway; 4 The CRANE collaboration, Bergen, Norway

**Keywords:** BPH, Elderly, Surgery, Complications

## Abstract

**Introduction:**

There are few studies evaluating the outcomes associated with transurethral resection of the prostate (TURP) in extremely elderly men. Our objective was to assess the safety and efficacy of this procedure in this special population.

**Methods:**

As part of an ethically approved study, retrospective review was carried out of all patients ≥ 85 years who underwent TURP between 2014 and 2023 at a tertiary centre. Data was collected on demographics including frailty status, operative data and complications. Treatment success was defined as catheter free status.

**Results:**

Over the study period, 194 patients (median age 87 years, IQR 86–89) underwent TURP, with a median follow-up of 6 years (IQR 3.5–8). The majority (68%) were ASA 3. 28% received home nursing assistance or were nursing home residents at the time of surgery. Median CCI score was 2 (IQR 1–3). 66% used anticoagulant medication. 97% received spinal anaesthesia and median operative time was 63 min (IQR 39–88). The intra-operative complication rate was 2.6%. The 30-day complication rate was 30% (Clavien-Dindo (CD) 1–2: 24%, ≥ CD 3: 5.7%). Among those with a catheter preoperatively (62%, n = 120), 84% had achieved spontaneous voiding (n = 101) at three month follow up. Overall, 93% were still alive at 1 year post surgery. The cumulative reoperation rate during study follow up was 7.2% at 1 year, 10.9% at 3 years, and 11.5% at 5 years.

**Conclusion:**

Treatment success for TURP in extremely elderly men (≥ 85 years) is high, but the associated morbidity burden warrants the need for an individualised approach when considering men in this special population who are potential candidates for surgery.

## Introduction

Benign prostatic obstruction (BPO) is a common condition in older men, frequently leading to lower urinary tract symptoms (LUTS), acute urinary retention (AUR), and, in many cases, long-term catheter dependence [1]. With global life expectancy steadily increasing, the number of men aged ≥ 85 years presenting with symptomatic BPO is rising, which arguably poses new management challenges for urologists [2–4].

Transurethral resection of the prostate (TURP) remains the reference intervention for the surgical management of BPO [5]. While efficacy and morbidity profiles are well established in the general adult population, relatively few studies have examined outcomes specifically in the “extreme elderly”, i.e. men aged ≥ 85 years. Moreover, it is often the case that studies on these methods exclude the very elderly or include them only as a small subgroup [6]. However, it is a demographic that is becoming increasingly visible in clinical practice and a conservative approach may often be preferred due to concerns about frailty, anesthetic risk, and postoperative complications. Frailty, in particular, has been associated with adverse functional outcomes following TURP [7]. However, a non-surgical approach must be weighed against the substantial morbidity and reduced quality of life associated with long-term catheterization, including reduced mobility, urinary tract infections, hematuria, and significant psychological burden [8].

In addition to LUTS and AUR, many elderly men with BPO experience recurrent macroscopic hematuria, often exacerbated by anticoagulant use [9]. Others develop recurrent bladder stones that cannot be definitively treated without addressing the underlying obstruction [10]. These factors can further contribute to patient morbidity [11].

The lack of evidence provides limited guidance to clinicians when assessing elderly patients with BPO and determining their suitability for surgery. In particular, it remains limited regarding how common preoperative characteristics—such as catheter dependency, anticoagulation use or, frailty status affect outcomes in men aged ≥ 85 [12].

The present study aims to address this knowledge gap by conducting a retrospective cohort study evaluating the safety, effectiveness, and postoperative outcomes of TURP in men aged ≥ 85 years. Specifically, we examine catheter-free rates, complication profiles, and one-year survival, and explore predictors of surgical success.

## Methods

### Study design, ethics and patient selection

We conducted a retrospective cohort study of male patients aged ≥ 85 years who underwent transurethral resection of the prostate (TURP) between 2014 and 2023 at a referral center for BPO surgery in Western Norway. During the study period, TURP was the only endoscopic treatment available, and minimally invasive surgical therapies (MISTs) were not in clinical use locally. The study was approved by the Regional Committee for Medical and Health Research Ethics (REK-no: 739374) and was conducted in accordance with the ethical principles outlined in the Declaration of Helsinki and its subsequent amendments. In compliance with the requirements set forth by the ethics committee, a negative (passive) informed consent process was employed: participants were informed about the study in writing and were included unless they explicitly declined participation. Data collection proceeded unless patients actively declined (n = 2) within four weeks. No external funding was obtained.

### Data collection and outcome definitions

Medical records of included patients were reviewed, and variables collected included age, American Society of Anesthesiologists (ASA) physical status classification, Charlson Comorbidity Index (CCI), catheter status, use of anticoagulant or antiplatelet medication, type of anesthesia, and operative details and mortality status. In lieu of formal frailty assessments, frailty status was inferred from care dependency, defined as receipt of formal home care assistance or nursing home residency. The primary outcome of interest was postoperative complications within 30 days, classified according to the Clavien–Dindo (CD) system. A secondary outcome was treatment success, defined as catheter-free status at 3-month follow-up.

### Pre-operative assessments

Standard pre-operative assessments included physical examination, uroflowmetry, and cystoscopy. In those with a history of bladder outlet obstruction surgery, cystoscopy confirmed recurrent obstruction. Prostate volume was most commonly assessed by transrectal ultrasound; however, alternative imaging modalities, such as magnetic resonance imaging, were also used when available. Urodynamic studies were not routinely performed.

### Surgical technique and catheter care

All procedures were performed using a standardized bipolar resection technique with the TURis system (Olympus Medical Systems, Tokyo, Japan), in accordance with institutional protocols. Operations were carried out either by experienced urologists specializing in endoscopic management of BPO or by residents under direct supervision. Catheters placed intraoperatively were removed before hospital discharge.

### Perioperative antithrombotic management

Perioperative antithrombotic management followed national recommendations [13]. Direct oral anticoagulants were withheld for 2 days, clopidogrel/ticagrelor for 5 days, and warfarin until INR < 1.6. Aspirin was discontinued only when used for primary prophylaxis. Bridging was considered in high-risk cases after consultation with a hematologist and/or cardiologist. Antithrombotic agents were restarted once the surgeon deemed the urine sufficiently clear.

### Statistical analysis

Logistic regression analyses were performed to identify predictors of complications and treatment failure for patients that were catheter dependent pre-operatively. Kaplan–Meier survival curves were generated to compare mortality against estimated data for men with same age from Norwegian reference population [14]. All statistical analyses were conducted using R (R Foundation for Statistical Computing, Vienna, Austria).

## Results

### Patient characteristics

We included 194 TURP procedures performed in patients aged ≥ 85 years with a median follow up of 6 years (IQR 3.5–8). The preoperative and intraoperative characteristics are summarized in Table 1. The median age was 87 years (IQR 86–89). Most patients were classified as ASA III (68%, n = 131), and the median CCI score was 2 (IQR 1–3). A total of 28% (n = 55) received formal care assistance at home care or were nursing home residents. Anticoagulant or antiplatelet therapy was used by 66% (n = 128). Preoperative catheterization was present in 62% (n = 120). Baseline prostate volume was available for 144 patients (74%), with a median of 58 mL (IQR 35–90).

**Table 1:** Summary of demographics

Total number of patients	194
Total number of TURP procedures	194
Median age (IQR)	87 years (86—89)
ASA grade	
1	1 (0.5%)
2	54 (28%)
3	131 (68%)
4	8 (4.1%)
Median pre-operative hemaglobin (IQR)	13.5 g/dL (IQR 12.4–14-4)
Median CCI (IQR)	2 (1–3)
Living status:	
Lives at home without public assistance	139 (72%)
Lives at home with home nursing care	51 (26%)
Nursing home residence	4 (2.1%)
Catheter status	
None	74 (38%)
Indwelling catheter	63 (33%)
Clean intermittent catheterization	50 (26%)
Suprapubic catheter	7 (3.6%)
Anticoagulant/platelet inhibitor use	128 (66%)
Previous BPO surgery	44 (23%)
Previous bladder stone surgery	7 (3.6%)
Median preoperative PSA (IQR)	5.2 (2.0–10.4)
Median prostate volume (IQR) (n = 144)	58 ml (35–90)
Preoperative urine culture (n = 173):	
No growth	104 (60%)
Growth in urine culture	69 (40%)

TURP = transurethral resection of the prostate

IQR = interquartile range

ASA = American Society of Anesthesiologists

CCI = Charlson Comorbidity Index

BPO = benign prostatic obstruction

LUTS = lower urinary tract symptoms

UTI = urinary tract infection

PSA = prostate specific antigen

Among patients without a catheter prior to TURP (n = 74), the main indications for surgery were persistent LUTS (n = 62, 84%), recurrent urinary tract infection (n = 10, 14%), and hematuria (n = 2, 3%). In the overall cohort (n = 194), 131 patients (67.5%) received some form of medical therapy prior to surgery, while 63 patients (32.5%) had not received any. The most common regimen was combination therapy with an α-blocker and 5-ARI (n = 98, 50.5%), followed by α-blocker monotherapy (n = 19, 9.8%) and 5-alpha reductase inhibitor monotherapy (n = 14, 7.2%).

### Intraoperative and perioperative data

Spinal anesthesia was used in 97% of procedures (Table 2). The median operative time was 63 min (IQR 39–88), and the median resected tissue weight was 26 g (IQR 13–45). Intraoperative complications occurred in 2.6% and consisted of bleeding from the prostate (1%), bleeding related to suprapubic drainage (0.5%), angina pectoris (0.5%), and inadvertent resection of the ureteral orifice (0.5%). The transfusion rate was 5.7%. The median decrease in hemoglobin on postoperative day 1 was 1.1 g/dL (IQR 0.6–1.7). The median hospital stay was 1 day (IQR 1–2).

**Table 2:** Operative data

Anesthesia type	
General anesthesia	6 (3.1%)
Spinal	188 (97%)
Median operative time (minutes) (IQR)	63 (39—88)
Median resected weight (gram) (IQR)	26 (13—45)
Operator experience	
Consultant urologist	116 (60%)
Resident	78 (40%)
Median hospital stay (days) (IQR)	1 (1—2)
Median change in hemoglobin (g/dL) (IQR)	1.1 (0.6 – 1.7)

IQR = interquartile range

### Complications

Postoperative complications within 30 days occurred in 30% (Table 3). Most were CD 1–2 (24%) while CD ≥ 3 complications occurred in 5.7%. One mortality was recorded and consisted of in hospital cardiac arrest at 24 h postoperatively. There was one death, which resulted from an in-hospital cardiac arrest 24 h postoperatively.

**Table 3:** Summary of complications

Early postoperative complications within 30 days	Management	CD	
Urinary retention	Catheter	II	10 (5.2%)
Urinary tract infection	Oral antibiotics	II	15 (7.7%)
Urinary sepsis	IV antibiotics + vasopressors	IV	1 (0.5%)
Hematuria	Irrigation catheter ± transfusion	II	14 (7.2%)
Hematuria	Cystoscopic clot evacuation (sedation/local)	IIIa	3 (1.5%)
Hematuria	Cystoscopic clot evacuation (spinal anesthesia)	IIIb	1 (0.5%)
Non–ST–Elevation myocardial infarction*	LMWH + DAPT	II	1 (0.5%)
Non–ST–Elevation myocardial infarction*	LMWH + DAPT + chest tube (local)	IIIa	1 (0.5%)
Atrioventricular block	Observation + beta-blocker withdrawal	I	1 (0.5%)
Atrioventricular block	Pacemaker (local)	IIIa	1 (0.5%)
ST–Elevation myocardial infarction*	PCI (local)	IIIa	1 (0.5%)
Transient ischemic attack	DAPT (dipyridamole + ASA/clopidogrel)	II	2 (1.0%)
Concussion after fall	Observation + wound suturing (local)	IIIa	1 (0.5%)
Acute psychiatric episode	Psychiatric pharmacotherapy	II	1 (0.5%)
Cerebral infarction *	Thrombolysis	II	1 (0.5%)
Acute renal failure	IV fluid therapy	II	2 (1.0%)
Bleeding gastric ulcer	Endoscopic hemostasis + transfusion	IIIa	1 (0.5%)
Death (cardiac arrest)	–	V	1 (0.5%)
Complication rate			
CD I-II			47 (24%)
CD ≥ III			11 (5.7%)
Overall			58 (30%)

^*^Note that these cases did not require ICU admission

CD = Clavien–Dindo

IV = Intravenous

LMWH = Low molecular weight heparin

DAPT = Dual antiplatelet therapy

PCI = Percutaneous coronary intervention

ASA = Acetylsalicylic acid

On logistic regression no significant predictors were identified for postoperative complications. On logistic regression, no significant predictors of postoperative complications were identified. Variables tested included age, ASA score, operative time, resection weight, and preoperative catheter status.

### Functional outcomes

At 3-month follow-up, 90% (n = 173) of patients were catheter-free; none required reoperation within this period to achieve catheter-free status. Among patients with an indwelling catheter preoperatively (62%, n = 120), 84% (n = 101) achieved spontaneous voiding. In this subgroup, lower resection weight was the only significant predictor of treatment failure (OR 0.94, 95% CI 0.90–0.99, p = 0.01), whereas age, ASA score, and operative time were not significant predictors. Paired comparisons among patients without a preoperative catheter (n = 74) demonstrated significant improvements following TURP. Qmax increased from a preoperative median of 8 ml/s (IQR 6–11) to a postoperative median of 14 ml/s (IQR 10–21) (*p* < 0.001). Similarly, PVR was significantly reduced, from a preoperative median of 90 ml (IQR 40–138) to a postoperative median of 22 ml (IQR 0–67) (*p* < 0.001, Wilcoxon signed-rank test).

Among men who were catheter free, the reported urinary incontinence rate was 6.9% (n = 12).

### Long-term outcomes

**Fig. 1:**
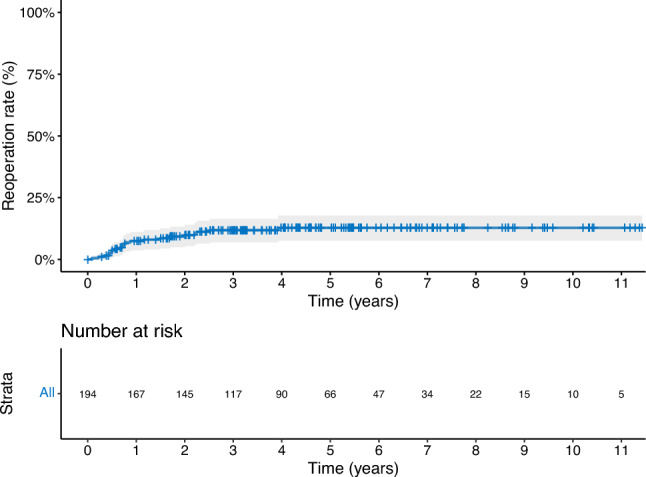
Cumulative rate of reoperation

**Fig. 2:**
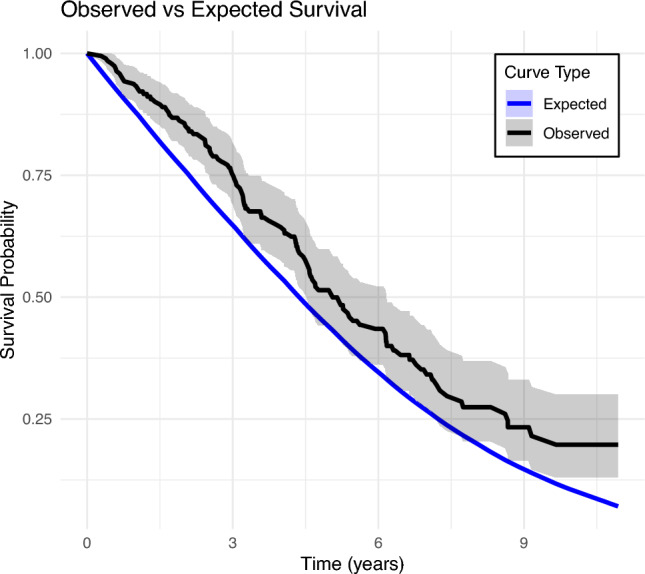
Overall survival of study sample compared to expected survival for Norwegian reference population

The median overall time to reoperation was 8.8 months (IQR 5.8–19.8). The cumulative reoperation rate during study follow-up was 7.2% at 1 year, 10.9% at 3 years, and 11.5% at 5 years (Fig. 1, Table 4). Reoperations consisted of re-TURP (4.1%, n = 8), bladder neck incision (5.7%, n = 11), cystolitholopaxy (1.0%, n = 2), and internal urethrotomy (0.5%, n = 1). One-year survival was 93%. This was not worse than the expected survival for men of the same age in the general Norwegian population (Fig. 2).

**Table 4:** Follow up data

Catheter status	
Catheter free	173 (90%)
Indwelling catheter	8 (4.2%)
Clean intermittent catheterization	12 (6.2%)
Histology	
Benign	129 (67%)
Malignant	63 (33%)
Mortality status:	
Death within 30 days	1 (0.5%)
Death within 1 year	13 (6.7%)
Elective surgical retreatment during study follow up	22 (11%)

## Discussion

This study confirms that TURP is an effective treatment option for bladder outlet obstruction in the extremely elderly. Catheter-free status was achieved in the vast majority of patients, and the rate of major complications (Clavien–Dindo ≥ 3) was low. Overall, these results support the premise that chronological age alone should not preclude patients from BPO surgery. These findings are consistent with a multicenter study by Lotterstätter et al., who similarly found TURP to be effective in selected patients aged ≥ 85 years [15]. In their study, the authors only identified polypharmacy to be a risk factor for failure to void spontaneously.

These favorable outcomes are consistent with those reported in a recent Holmium Laser Enucleation of the Prostate (HoLEP) series from Klein et al. [16]. Similarly, in a multicenter study by Priola et al., which included 731 patients with a median age of 82 years undergoing endoscopic enucleation, the major complication rate was low at 2.2% [17]. In addition, in a large study of 981 patients stratified by age 75 years and above versus below 75 years, Giulioni et al., found that functional outcomes were comparable at 1 year follow-up [18]. Photoselective Vaporization of the Prostate (PVP) is also a potential alternative and could offer advantages proposed for patients on anticoagulation or with an elevated anesthetic risk [19]. In a study by Macdonald et al., 57 octogenarians underwent PVP, with 91% classified as ASA III and 7% as ASA IV [20]. The overall complication rate was 10.5% and 93% were catheter free at one year follow up.

The rate of cumulative reoperations plateaued after 3 years. This could be interpreted in several ways. It could infer that most reoperations are necessitated early on and/or that if a patient reaches this time point, their outcomes are durable. Alternatively, the rate could artificially flatten beyond this time period as patients may become too frail to be considered eligible for repeat surgery and a conservative method is adopted accordingly. It is also noteworthy that the one-year survival in our cohort was 93%, similar to a matched age group based on national demographic data [21]. This most likely reflects selection bias and is a common finding in such studies in elderly populations. Outcome data including survival data on those who were denied surgery based on a high-risk profile would be valuable. Nonetheless, our findings support offering definitive treatment to appropriately selected patients in this age group. It also supports avoiding rigid age-based criteria in favor of a more nuanced, holistic assessment of the patient. Ideally, this should involve a multidisciplinary approach, incorporating surgical, anesthesiological, and geriatric evaluation, with the aim of minimizing intra-, peri-, and postoperative complications. While ASA and CCI scores are helpful, the integration of established geriatric tools—such as gait speed, cognitive status, and polypharmacy burden can further refine surgical risk stratification [22, 23].

MISTs are also a therapeutic alternative although data in the ≥ 85 age group are more limited. Furthermore, access to newer surgical modalities remains limited in smaller nations such as Norway. According to national registry data, in 2023 TURP constituted over 90% of BPO procedures performed, laser enucleation accounted for 1.1%, and the remainder were simple prostatectomies conducted via either open or robotic approaches [24]. MISTs only became available in Western Norway after the conclusion of the study period. Accordingly, the findings presented here reflect outcomes from a context in which TURP was the sole endoscopic treatment option.

MISTs that have been developed for the treatment of BPO include transperineal laser ablation (TPLA), iTind, Rezūm, and urolift [25–28]. Many of these such as TPLA can also be performed under local anaesthesia [29]. Destefanis et al., reported outcomes of TPLA in high-risk surgical patients, demonstrating significant improvements in both prostate volume reduction and IPSS scores [30]. Data on MIST outcomes in catheter-dependent cohorts and in the very elderly remain limited, and available studies suggest lower rates of achieving catheter-free status in patients with a long-term catheter patients [31, 32].

The overall reoperation rate of 11% in our cohort is higher than the pooled 5 year retreatment rate of 7.7% reported by He et al. in their systematic review of TURP outcomes, although still lower than the corresponding rate reported for prostate artery embolization (23.8%) [33]. This discrepancy could reflect differences in patient selection, follow-up duration, or reoperation definitions across studies. This could include for example if the definition for reoperation only refers to repeat TURP. Surgeons may also adopt a more conservative approach in higher-risk patients, performing more limited resections and thereby increasing the likelihood of recurrent obstruction. Furthermore, factors common in this age group, such as prolonged preoperative catheterisation, comorbidities like diabetes, and reduced vascular supply, may predispose to fibrosis and the development of complications such as bladder neck contracture.

Similar to the study by Macdonald et al., a small proportion of patients in our cohort (n = 8, 4.1%) had a high comorbidity burden (ASA IV) [20]. Although such patients carry significant perioperative risk, they were cognitively intact, expressed strong wishes to be free of a catheter, and were carefully counselled before surgery. For these individuals, the potential benefits included avoidance of chronic catheter-related complications, preservation of independence, and the possibility of improving quality of life despite limited life expectancy [34, 35].

## Strengths and limitations

This study is limited by its retrospective, single-center design and lack of baseline prostate volume in all patients. While catheter-free status is a pragmatic endpoint, subjective outcomes such as IPSS were not available. As such, reliable conclusions cannot be drawn regarding the impact of TURP on patients’ quality of life, sexual function or broader patient related outcomes. The cohort consisted only of patients deemed fit for surgery, which may introduce selection bias. This likely contributed to the high one-year survival rate. Accordingly, the findings may not be fully generalizable to the broader population of very elderly men with LUTS or urinary retention. Frailty was not formally assessed using a standardized instrument, which limits the precision of frailty assessment. Use of validated tools such as the Clinical Frailty Scale (CFS) or G-8 Geriatric Screening Tool would have provided a more robust evaluation of frailty, which represents a multidimensional and complex phenotype [36, 37].

Despite these limitations, the study provides real-world reference data in a growing demographic for which evidence remains scarce. Future studies should prospectively validate frailty-based risk models, and the development of a dedicated clinical decision tool could aid preoperative counseling in this special population. This could be complemented by qualitative research exploring to what extent relief from catheter dependence may improve quality of life, particularly when patients weigh this potential benefit against the risks of surgery. Comparative trials between TURP and alternative surgical approaches in men ≥ 85 years are warranted, and parallel health-economic analyses may clarify the cost-effectiveness of TURP compared with long-term catheterization.

## Conclusion

TURP in men aged ≥ 85 years is effective, including in catheter-dependent patients, and is associated with a low rate of serious adverse events. Chronological age alone should not be regarded as a contraindication; rather, surgical decisions should be made in the context of an individual patient’s comorbidity profile and functional status.

## Data Availability

. Available upon reasonable request to the corresponding author.
